# First high-quality genome assembly of *Umbelopsis nana* isolated from forest soil

**DOI:** 10.1093/g3journal/jkag022

**Published:** 2026-01-29

**Authors:** Ryuka Iizuka, Tomohiro Suzuki, Nozomu Watanabe, Javier F Tabima, David Hibbett, Yoko Katayama, Makoto Yoshida

**Affiliations:** Institute of Global Innovation Research, Tokyo University of Agriculture and Technology, 3-5-8 Saiwai-cho, Fuchu-shi, Tokyo 183-8509, Japan; Center for Bioscience Research and Education, Utsunomiya University, 350 Mine-Machi, Utsunomiya-shi, Tochigi 321-8505, Japan; Center for Bioscience Research and Education, Utsunomiya University, 350 Mine-Machi, Utsunomiya-shi, Tochigi 321-8505, Japan; Department of Biology, Clark University, Worcester, MA 01610, United States; Department of Biology, Clark University, Worcester, MA 01610, United States; Institute of Agriculture, Tokyo University of Agriculture and Technology, 3-5-8 Saiwai-cho, Fuchu, Tokyo 183-8509, Japan; Independent Administrative Institution, Tokyo National Research Institute for Cultural Properties, 13-43 Ueno-Park, Taito-ku, Tokyo 110-8713, Japan; Institute of Global Innovation Research, Tokyo University of Agriculture and Technology, 3-5-8 Saiwai-cho, Fuchu-shi, Tokyo 183-8509, Japan; Institute of Agriculture, Tokyo University of Agriculture and Technology, 3-5-8 Saiwai-cho, Fuchu, Tokyo 183-8509, Japan

**Keywords:** *Umbelopsis nana*, genome, PacBio, phylogenetic analysis, functional annotation, forest ecosystem, lipid metabolism, genome assembly

## Abstract

Species of the genus *Umbelopsis* are ubiquitous soil saprotrophic fungi known for their role in ecological nutrient cycling and biotechnological potential in lipid production. Here, we present the genome assembly of *Umbelopsis* sp. strain THIF13, which is characterized by its capability to produce carbonyl sulfide. Using PacBio Sequel long-read data, a high-quality genome assembly was generated comprising 18 contigs totaling 27.2 Mb, which is larger than other *Umbelopsis* genomes, with an N50 of 1.77 Mb. Notably, 16 contigs were flanked by telomeric repeats at both ends. The assembly showed high completeness, with 98.1% complete Benchmarking Universal Single-Copy Orthologs and 11.15% repetitive sequences. Multilocus phylogenetic analysis clearly identified the strain as *Umbelopsis nana*; thus, this study reports the first publicly available genome sequence for this species. Functional annotation with Funannotate predicted 9,068 genes. This genomic resource fills a gap in the *Umbelopsis* phylogeny and provides a foundation for elucidating the evolutionary and functional diversity of the genus.

## Introduction

Species of the genus *Umbelopsis* (order Umbelopsidales, subphylum Mucoromycotina) are ubiquitous soil saprotrophs that contribute to lignocellulosic biomass decomposition and nutrient cycling in forest ecosystems. These fungi inhabit terrestrial environments and are primarily isolated from forest soil, but they can also be isolated from the fruiting bodies of mushrooms (Parshikov et al. 1999; Watanabe et al. 2001; Summerbell 2005). Although their ecological role as decomposers in forest ecosystems is well recognized, certain species within *Umbelopsis*, such as *U. isabellina* and *U. ramanniana*, have garnered significant interest for their biotechnological potential, particularly in lipid production (Kamisaka et al. 1993; Pillai et al. 1998; Chatzifragkou et al. 2010; Papanikolaou and Aggelis 2019). The lipids they accumulate are rich in polyunsaturated fatty acids (Gardeli et al. 2017; Hou et al. 2024), making them attractive candidates for sustainable biodiesel and nutritional applications.

*Umbelopsis* previously included species assigned to *Mortierella* or *Micromucor* (Mortierellales) but is now classified in its own order, Umbelopsidales (Amos and Barnett 1966; Yip 1986; Spatafora et al. 2016). Historically, species delimitation in *Umbelopsis* largely depended on morphological traits such as sporangial structure and colony appearance. However, the subtle variations and often overlapping characteristics among species made it difficult to achieve clear identification based on morphology alone (Meyer and Gams 2003; Sugiyama et al. 2003). Early molecular investigations in *Umbelopsis* primarily utilized nuclear ribosomal DNA loci, including the internal transcribed spacer (ITS) region and large subunit (LSU) rRNA genes (Meyer and Gams 2003; Sugiyama et al. 2003). However, subsequent research increasingly demonstrated that relying on a single ribosomal DNA marker, such as ITS alone, often lacked the resolution needed for reliable species-level identification and conflicted with morphological data in *Umbelopsis* (Ogawa et al. 2005, 2011). To address these limitations, protein-coding genes such as actin (*ACT*) were added to multilocus sequence analyses. The combined use of ribosomal and protein-coding markers (ITS-LSU-*ACT*) significantly improved resolution, enabling clearer species boundaries and facilitating the description of new taxa (Wang et al. 2014, 2015; Crous et al. 2017; Hou et al. 2024). Moreover, a comprehensive 6-gene phylogeny combining ribosomal DNA with protein-coding loci such as minichromosome maintenance complex component 7 (*MCM7*) and cytochrome c oxidase subunit I (*COX1*) proved instrumental in resolving the cryptic *U. ramanniana* sensu lato species complex (Wang et al. 2022).

Despite their recognized ecological importance, diverse application potential, and advances in phylogenetic understanding, comprehensive genomic resources for *Umbelopsis* species remain limited. To date, genome sequences have been reported for only a few representative *Umbelopsis* species (mainly *U. isabellina* and *U. ramanniana*), while biological and genomic information on other valid species, such as *Umbelopsis nana*, remains limited, focusing mainly on morphological and phylogenetic descriptions (e.g. Wang et al. 2015). In this study, we focused on *Umbelopsis* sp. strain THIF13, which was originally isolated from forest soil and characterized by its capability to produce carbonyl sulfide (COS), a major sulfur-containing gas in the atmosphere (Masaki et al. 2016). However, its precise species identity remained unresolved because initial identification relied solely on the ITS region, which offers insufficient resolution for differentiating closely related species within the genus. Therefore, a high-quality genome assembly was generated using the PacBio Sequel system and multilocus phylogenetic analysis was performed. These analyses identified the strain as *U. nana*; thus, this study provides the first genomic insight into this species, directly addressing the knowledge gap regarding its evolutionary and functional diversity.

## Materials and methods

### Mycelium cultivation and DNA sequencing

*Umbelopsis* sp. strain THIF13 (deposited in the NITE Biological Resource Center (NBRC), National Institute of Technology and Evaluation (NITE), under the accession number NBRC 117090) was originally isolated from forest soil collected in the Karasawa-yama area, Field Museum, Tokyo University of Agriculture and Technology, Tochigi, Japan, and subsequently established via single-spore isolation (Masaki et al. 2016). The strain was cultivated in potato dextrose broth (Becton, Dickinson and Company, Franklin Lakes, NJ) at 25 °C with shaking at 150 rpm for 12 d. Mycelial biomass was harvested by vacuum filtration through filter paper, frozen in liquid nitrogen, and lyophilized. Genomic DNA was extracted using a cetyltrimethylammonium bromide extraction protocol (Doyle and Doyle 1987). Residual RNA was removed by incubation with RNase A (Qiagen, Hilden, Germany). A genomic DNA library was constructed using the [3.0] PacBio Microbial Library kit (PacBio, Menlo Park, CA) and HiFi sequencing was performed on a PacBio Sequel IIe system (PacBio).

### Genome assembly, filtering, and evaluation

To evaluate the heterozygosity of the sequenced strain, a k-mer analysis was performed. The PacBio HiFi reads were used to count 21-mers using Jellyfish v2.3.1 (Marçais and Kingsford 2011), and the genome characteristics, including heterozygosity, were estimated using GenomeScope v2.0 (Vurture et al. 2017; Ranallo-Benavidez et al. 2020).

De novo assembly was performed with hifiasm v0.25.0-r726 (Cheng et al. 2021) (Supplementary File 1). Circular contigs were detected and redundant sequences removed using Circlator v1.5.5 (Hunt et al. 2015) (Supplementary File 2). These circular contigs were identified as the putative mitochondrial genome via BLAST searches and were separated from the nuclear assembly. To remove redundant contigs representing putative haplotigs or assembly artifacts, whole-contig alignment was first performed with Mauve v1.1.3 (Darling et al. 2004) to identify containment relationships. Subsequently, redundancy was quantified by contig-vs-contig BLASTN searches using BLAST+ v2.15.0 (Camacho et al. 2009). Contigs showing >99.9% identity over 90% of their length were excluded. Filtered contigs were then scanned for telomeric repeats using tidk v0.2.65 (Brown et al. 2025) within a 6,000 bp window at the contig ends (Supplementary File 3). Assembly continuity metrics, including N50, were calculated with N50Stat.pl from the NGS QC Toolkit v2.3 (Patel and Jain 2012) and assembly completeness was evaluated with Benchmarking Universal Single-Copy Orthologs (BUSCO) v5.7.1 against the fungi_odb10 dataset (Simão et al. 2015).

### Repeat sequence prediction

A species-specific repeat library was generated using RepeatModeler2 v2.0.6 (Flynn et al. 2020), which included the LTR structural discovery pipeline (-LTRStruct flag). The resulting repeat library was used with RepeatMasker v4.1.9 (Smit et al. 2015), utilizing RMBlast 2.14.1+ (Camacho et al. 2009) as the search engine to identify and soft-mask repetitive elements throughout the genome, employing the -xsmall option to report only the most significant matches (Supplementary File 4).

### Gene prediction and functional annotation

Gene models were predicted using the Funannotate v1.8.17 pipeline (Palmer and Stajich 2020) (Supplementary File 5). First, the genome assembly, previously soft-masked for repetitive elements, was further soft-masked with Tantan v50 (Frith 2011) to optimize gene prediction. Conserved single-copy orthologs identified by BUSCO v5.7.1 (fungi_odb10 dataset) (Simão et al. 2015) were employed to train the ab initio predictors Augustus v3.3.2 (Stanke and Morgenstern 2005), GlimmerHMM v3.0.4 (Majoros et al. 2004), and SNAP v2006-07-28 (Korf 2004), while GeneMark-ES v4.72 (Ter-Hovhannisyan et al. 2008) was self-trained on the assembly. Transcript and protein evidence from *U. ramanniana* strain AG (Amses et al. 2022), comprising RNA-seq-derived transcripts and annotated protein models downloaded from the JGI MycoCosm portal (Grigoriev et al. 2012, 2014), were aligned to the masked genome. The transcripts were aligned using Minimap2 v2.28-r1209 (Li 2018) to generate an exon-intron hints file. Protein evidence was incorporated by mapping the protein set to the genome, first with a rapid search using DIAMOND v2.1.8 (Buchfink et al. 2015) followed by a more precise alignment with Exonerate v2.4.0 (Slater and Birney 2005) to refine gene structure predictions. Augustus was then run in evidence-aware mode using the hints file, with its parameters further optimized using the –optimize_augustus flag, while GeneMark-ES, GlimmerHMM and SNAP performed pure ab initio predictions. Finally, all evidence-based and ab initio outputs were merged into a nonredundant consensus gene set using EvidenceModeler v1.1.1 (Haas et al. 2008).

The consensus protein set was annotated with InterProScan v5.69–101.0 (Jones et al. 2014) to assign domains, families, and Gene Ontology terms, and with eggNOG-mapper v2.1.12 (Cantalapiedra et al. 2021) using the eggNOG 5.0 database (Huerta-Cepas et al. 2019) via DIAMOND blastp for orthology (Buchfink et al. 2015), COG categories, and pathway mappings. These annotations were imported into Funannotate's annotate module together with the predicted gene models. Funannotate then performed HMMER searches against Pfam v37.0 (Mistry et al. 2021) and dbCAN v12.0 (Zhang et al. 2018), DIAMOND blastp searches against UniProt release 2024_04 (UniProt Consortium 2025) and MEROPS v12.5 (Rawlings et al. 2018), and BUSCO Dikarya model scans, and it standardized gene and product names with Gene2Product v1.94. Furthermore, Kyoto Encyclopedia of Genes and Genomes (KEGG) pathway annotation was retrieved from the eggNOG-mapper v2.1.12 results.

### Comparative analyses

For comparative analyses, genome and protein sequence data for *U. isabellina* strain WA0000067209 (BioProject: PRJNA668042) (Muszewska et al. 2021), and genome, transcript, and protein sequence data for *U. ramanniana* strain AG (BioProject: PRJNA196032) (Amses et al. 2022) were downloaded from the National Center for Biotechnology Information (NCBI) GenBank database. To ensure consistent annotation standards across all genomes, the repeat prediction workflow and the Funannotate pipeline described above were applied to these datasets.

### Phylogenetic analyses

6 loci (SSU rDNA, ITS, LSU rDNA, *ACT*, *MCM7*, and *COX1*) were retrieved from GenBank for 49 *Umbelopsis* strains. These sequences were concatenated with the sequence of *Umbelopsis* sp. strain THIF13, resulting in a dataset comprising 50 *Umbelopsis* strains for analysis. Two *Mortierella* species were added as outgroups (Table 1). For *Umbelopsis* sp. strain THIF13, the corresponding sequences for these 6 loci were extracted from its genome assembly by BLASTN searches using BLAST + v2.15.0 (Camacho et al. 2009), as whole-genome data were unavailable for most reference strains. Each locus was aligned independently with MAFFT v7.481 using the L-INS-i algorithm (Katoh and Standley 2013) (Supplementary File 6). The resulting alignments were then subjected to trimming with trimAl v1.4.rev15 using the -gappyout method to remove gappy regions based on automated thresholds (Capella-Gutiérrez et al. 2009). The 6 alignments were concatenated, and a RAxML-style partition file defining locus boundaries was generated. Maximum-likelihood inference (ML) was carried out with IQ-TREE 2 v2.4.0 (Minh et al. 2020), using optimal models for each partition as determined by ModelFinder (Kalyaanamoorthy et al. 2017): TN + F + R2 for SSU, HKY + F + R2 for *COX1*, HKY + F + I + G4 for *ACT*, TIM2 + F + G4 for *MCM7*, TIM3 + F + I + G4 for LSU, and TIM2 + F + G4 for ITS. Node support was evaluated via 1,000 ultrafast bootstrap replicates (Minh et al. 2013; Hoang et al. 2018) and 1,000 SH-like approximate likelihood ratio tests (Anisimova et al. 2011). During each replicate the tree was further optimized by likelihood-based nearest neighbor interchange to refine branch lengths and reduce artefactual support.

**Table 1.** Species, strains, and GenBank accession numbers of loci used in the phylogenetic analysis.

Species	Strains	SSU	ITS	LSU	*ACT*	*MCM7*	*COX1*
*M. minutissima*	CBS 277.71	EU736292	HQ630324	EU736319	EU736238	…	…
*M. verticillata*	NRRL 6337	AB016017	AY997063	DQ273794	AJ287170	…	…
U. angularis	CGMCC 3.664	KM017656	KC489475	KF727440	KF771831	MF417299	MF417175
*U. angularis*	CGMCC 3.6639	KM017657	KC815992	KM017696	KM017712	MF417300	MF417176
*U. angularis*	CGMCC 3.1634	MF417062	KC816008	MF417098	MF417147	MF417335	MF417209
*U. angularis*	CBS 603.68^HT^	NG_070102	KC489476	KF727442	KF771842	MF417301	MF417178
*U. changbaiensis*	CGMCC 3.16347	KM017672	KC489483	KF727446	KF771838	MF417318	MF417194
*U. curvata*	CGMCC 3.6647	MF417070	MF417290	MF417115	MF417164	MF417347	MF417227
*U. curvata*	CBS 219.47^HT^	MF417073	MF417293	MF417120	MF417169	MF417357	MF417237
*U. dimorpha*	CBS 110039^HT^	KM017689	KC489478	KF727471	KF771833	MF417365	MF417247
*U. dimorpha*	CGMCC 3.16325	KM017661	KC815993	KM017697	KM017725	MF417302	MF417180
*U. dimorpha*	CGMCC 3.16329	KM017665	KC815997	KM017701	KM017719	MF417311	MF417190
*U. dimorpha*	CGMCC 3.1633	KM017666	KC815998	KM017702	KM017720	MF417312	MF417191
*U. dimorpha*	CGMCC 3.16331	KM017667	KC815999	KM017703	KM017721	MF417313	MF417192
*U. dimorpha*	CGMCC 3.16332	KM017668	KC816000	KM017704	KM017722	MF417314	MF417193
*U. dura*	CGMCC 3.15777^HT^	MF417063	MF417275	MF417099	MF417148	MF417336	MF417210
*U. dura*	CGMCC 3.15778	MF417064	MF417276	MF417100	MF417149	MF417337	MF417211
*U. dura*	CGMCC 3.15779	MF417065	MF417277	MF417101	MF417150	MF417338	MF417212
*U. dura*	CGMCC 3.1578	MF417066	MF417278	MF417102	MF417151	MF417339	MF417213
*U. dura*	CGMCC 3.15781	MF417067	MF417279	MF417103	MF417152	MF417340	MF417214
*U. fusiformis*	CBS 385.85^HT^	KM017683	KC489500	KF727463	KF771861	MF417359	MF417240
*U. gibberispora*	CBS 109328^HT^	KM017688	KC489479	KF727469	KF771832	MF417364	MF417246
*U. isabellina*	NRRL 62705	KM017693	KM017729	KM017709	KM017716	MF417372	MF417253
*U. isabellina*	NRRL 1757	KM017692	KM017728	KM017707	KM017715	MF417371	MF417252
*U. longicollis*	CBS 209.32^T^	KM017655	KM017727	JN940876	KM017711	MF417368	MF417249
*U. macrospora*	NRRL 5844^HT^	KM017694	KM017730	KM017710	KM017713	MF417375	MF417256
*U. microsporangia*	CGMCC 3.15769	MF417053	MF417266	MF417085	MF417134	MF417322	MF417196
*U. microsporangia*	CGMCC 3.1577	MF417054	MF417267	MF417086	MF417135	MF417323	MF417197
*U. microsporangia*	CGMCC 3.15771	MF417055	MF417268	MF417087	MF417136	MF417324	MF417198
*U. microsporangia*	CGMCC 3.15782	MF417071	MF417291	MF417117	MF417166	MF417350	MF417230
*U. nana*	CBS 858.68^T^	KM017686	KC489503	KF727467	KF771864	MF417362	MF417244
*U. nana*	CBS 150.81	KM017680	KC489496	KF727459	KF771851	MF417355	MF417234
*U. nana*	CBS 473.74	KM017684	KF765510	KF727464	KF771862	MF417360	MF417241
*U. nana*	CGMCC 3.16327	KM017662	KC815995	KM017698	KM017714	MF417303	MF417182
*U. nana*	CGMCC 3.16328	KM017664	KC815996	KM017699	KM017718	MF417310	MF417189
*U. nana*	CGMCC 3.16343	KM017678	KC816011	KM017700	KM017726	MF417351	MF417231
*U. oblongielliptica*	NRRL 1296	KM017676	KC489493	KF727456	KF771847	MF417373	MF417254
*U. ovata*	CBS 499.82^T^	KM017685	KC489501	KF727465	KF771863	MF417361	MF417242
*U. ramanniana*	CGMCC 3.15772	MF417056	MF417269	MF417088	MF417137	MF417325	MF417199
*U. ramanniana*	CGMCC 3.15773	MF417057	MF417270	MF417089	MF417138	MF417326	MF417200
*U. ramanniana*	CGMCC 3.15774	MF417058	MF417271	MF417090	MF417139	MF417327	MF417201
*U. ramanniana*	CGMCC 3.15775	MF417059	MF417272	MF417091	MF417140	MF417328	MF417202
*U. ramanniana*	CGMCC 3.15776	MF417060	MF417273	MF417092	MF417141	MF417329	MF417203
*U. ramanniana*	CGMCC 3.6646	MF417068	MF417288	MF417113	MF417162	MF417345	MF417225
*U. ramanniana*	CGMCC 3.16356	KM017677	KC489494	KF727457	KF771848	MF417348	MF417228
*U. ramanniana*	CGMCC 3.15783	MF417072	MF417292	MF417119	MF417168	MF417353	MF417233
*U. swartii*	CBS 868.85^T^	KM017687	KC489504	KF727468	KF771865	MF417363	MF417245
*U. vinacea*	CGMCC 3.16352	KM017675	KC489489	KF727452	KF771846	MF417344	MF417224
*U. vinacea*	CBS 212.32^ET^	NG_063020	KC489498	KF727461	KF771853	MF417356	MF417236
*U. vinacea*	CBS 236.82	MF417074	KC489499	KF727462	KF771859	MF417358	MF417239
*U. westeae*	CBS 870.85^T^	KM017690	KC489505	KF727472	KF771834	MF417366	MF417248
*Umbelopsis* sp.	THIF13	LC848287	LC848289	LC848288	LC883923^a^	LC883924^a^	LC885049^a^

^a^The *ACT*, *MCM7*, and *COX1* sequences are full-length but were trimmed to the conserved regions shared with other taxa for phylogenetic inference.

HT, ex-holotype strain; ET, ex-epitype strain; T, ex-type strain.

Additionally, a Bayesian inference (BI) analysis was conducted using MrBayes v3.2.7a (Ronquist et al. 2012) to corroborate the ML topology. The same concatenated alignment and partition file were used. The GTR + I + G model was applied, with all parameters unlinked across the 6 partitions. Two independent MCMC runs were performed for 10 million generations, sampling every 1,000 generations. Convergence was confirmed when the average standard deviation of split frequencies (Lakner et al. 2008) fell below 0.01 and the potential scale reduction factor (Gelman and Rubin 1992) for all parameters approached 1.0. The first 25% of samples (2,500 trees) were discarded as burn-in, and a 50% majority-rule consensus tree was generated from the remaining trees to obtain posterior probabilities. The resulting phylogenies (both ML and BI) were visualized in iTOL v7 (Letunic and Bork 2024).

## Results and discussion

### Genome assembly, filtering, and evaluation

Whole-genome sequencing was performed using the PacBio Sequel IIe system. A total of 113,886 HiFi reads were generated, with a mean read length of 7,752 bp and a read N50 of 9,012 bp. All HiFi reads were used for de novo assembly. Prior to assembly, a k-mer analysis was performed on these PacBio HiFi reads to evaluate genome characteristics, particularly heterozygosity. The 21-mer frequency spectrum showed a single, sharp peak (Supplementary Fig. 1a), which is characteristic of a haploid or homozygous genome. The estimated heterozygosity rate was 0 to 0.033% (Supplementary Fig. 1b). This result strongly indicates that *Umbelopsis* sp. strain THIF13 was monokaryotic, or at least highly homozygous.

**Fig. 1.**
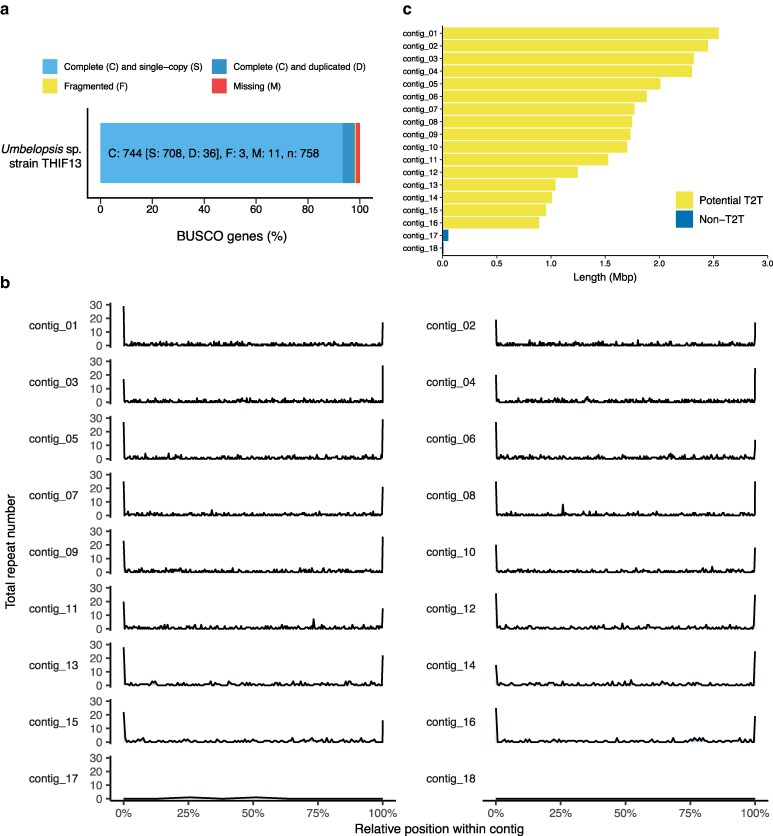
Evaluation of the *Umbelopsis* sp. strain THIF13 draft genome assembly. a) BUSCO completeness based on the fungi_odb10 dataset. b) Occurrence of the telomeric repeat “AAACCCT” across the 18 genomic contigs. Each panel shows an individual contig. The *x*-axis represents the normalized position within each contig, while the *y*-axis denotes the frequency of the identified repeat. c) Length distribution of the filtered contigs. Contigs with telomeric repeats detected at both ends (Potential telomere-to-telomere, Potential T2T) are shown in yellow; contigs lacking telomeric repeats (Non-T2T) are shown in blue.

To generate a high-quality draft genome of *Umbelopsis* sp. strain THIF13, PacBio HiFi reads were assembled with hifiasm, yielding 25 contigs. Nonchromosomal and redundant sequences were removed through a 2-step filtering process. In the first step, Circlator detected 2 circular contigs (putative mitochondrial genomes) and 2 additional contigs whose entire sequences were contained within other contigs, all of which were subsequently excluded. In the second step, the remaining 21 contigs were aligned with Mauve and subjected to contig–contig BLASTN search to detect sequences sharing > 99.9% identity. Three contigs identified as redundant were discarded, resulting in a final assembly of 18 contigs. BUSCO analysis against the fungi_odb10 dataset revealed 744 complete BUSCOs out of 758 groups (98.1%) both before and after filtering, indicating high assembly completeness and confirming that only duplicate sequences had been removed (Fig. 1a). Scanning with tidk identified the heptameric repeat AAACCCT. Visual inspection of the repeat density (Fig. 1b) revealed distinct peaks concentrated at both ends of the 16 contigs. This distinct localization pattern suggests high contiguity and potential chromosome completeness (Fig. 1c). However, definitive confirmation of the chromosomal structure requires further validation using complementary methods, such as Hi-C sequencing or karyotype analysis. Interestingly, while the hexamer TTAGGG telomere motif is commonly observed in filamentous fungi (Podlevsky et al. 2008), the AAACCCT repeat observed in *Umbelopsis* represents a lineage-specific divergence in telomere sequence. The final *Umbelopsis* sp. strain THIF13 assembly comprises 18 contigs totaling 27.2 Mb, with an N50 of 1.77 Mb and a median contig length of 1.72 Mb, indicative of a highly contiguous assembly (Table 2). Notably, the genome size of *Umbelopsis* sp. strain THIF13 is larger than previously reported *Umbelopsis* genomes, such as *U. ramanniana* AG (23.1 Mb) (Amses et al. 2022), *U. isabellina* (22.1 Mb), *U. vinacea* (23.2 Mb) (Muszewska et al. 2021), and *Umbelopsis* sp. WA50703 (22.7 Mb) (Dziurzyński et al. 2025).

**Table 2.** Assembly statistics for the filtered contigs.

	Value
Total contigs	18
Total assembly size (Mb)	27.2
Average sequence length (Mb)	1.51
Median sequence length	1.72
N50 length (Mb)	1.77
N25/N75/N90/N95 length (Mb)	2.32/1.70/1.01/0.95
L50	7
GC content (%)	44.16
Ambiguous bases (Ns) (%)	0.00
Total repetitive content (%)	11.15
Genome coverage	30×

### Repeat sequence annotation

Analysis of the filtered genome assembly revealed that 11.15% (3,032,892 bp) of the assembly was annotated as repetitive sequences. The majority of these were interspersed repeats (10.44%), predominantly composed of DNA transposons (4.59%) and unclassified elements (4.80%). Within DNA transposons, the Tc1-IS630-Pogo superfamily was the most prevalent, dominating the repeat landscape in *Umbelopsis* sp. strain THIF13. Retroelements comprised 1.05% of the genome, with LTR elements (0.82%, mainly Gypsy/DIRS1) and LINEs (0.23%, mainly L1/CIN4) being identified. Other repeat categories included simple repeats (0.51%), low complexity regions (0.08%), and small RNA-related sequences (0.12%).

To investigate the larger genome size (27.2 Mb) of strain THIF13, a comparative analysis was performed with *U. ramanniana* and *U. isabellina*. The reference species exhibited markedly lower repeat contents (1.65% and 0.90%, respectively) compared with *Umbelopsis* sp. strain THIF13 (11.15%). These results suggest that the genomic expansion in *Umbelopsis* sp. strain THIF13 is likely attributable to the accumulation of repetitive elements and the associated increase in noncoding intergenic regions, given that the number of functional genes remains conserved.

### Functional annotation

Gene prediction and functional annotation were performed for the *Umbelopsis* sp. strain THIF13 genome and the annotation metrics were compared with 2 representative *Umbelopsis* species, *U. isabellina* and *U. ramanniana* (Table 3). The *Umbelopsis* sp. strain THIF13 genome was predicted to contain a total of 9,068 genes. While this is slightly higher than *U. isabellina* (8,839 genes), it is lower than *U. ramanniana* (9,213 genes). Overall, a substantial proportion of genes were successfully annotated across all 3 *Umbelopsis* genomes, with 84.2% annotated by InterProScan, 89.6% by eggNOG, 74.5% by GO-term, and 76.6% by Pfam for *Umbelopsis* sp. strain THIF13. Regarding specific functional categories, 3.9% of *Umbelopsis* sp. strain THIF13 genes were annotated as carbohydrate-active enzymes (CAZymes) and 3.5% as MEROPS peptidases. Such variations may also be influenced by differences in sequencing technologies and assembly approaches.

**Table 3.** Comparative functional annotation metrics for *Umbelopsis* sp. strain THIF13, *U. isabellina*, and *U. ramanniana*.

Category	*Umbelopsis* sp. strain THIF13		*U. isabellina*		*U. ramanniana*	
InterProScan-annotated proteins	7,635	(84.2%)	7,482	(84.6%)	7,838	(85.1%)
eggNOG-annotated proteins	8,129	(89.6%)	7,909	(89.5%)	8,311	(90.2%)
GO-term-assigned genes	6,753	(74.5%)	6,647	(75.2%)	6,950	(75.4%)
Pfam-annotated genes	6,946	(76.6%)	6,877	(77.8%)	7,241	(78.6%)
CAZyme-annotated genes	356	(3.9%)	340	(3.8%)	343	(3.7%)
MEROPS-annotated genes	317	(3.5%)	319	(3.6%)	320	(3.5%)
BUSCOs identified in annotation	1,291	(14.2%)	1,287	(14.6%)	1,356	(14.7%)
Total genes	9,068	…	8,839	…	9,213	…

The comparable proportions of CAZymes and MEROPS peptidases, along with the overall similarity in annotation metrics across the 3 *Umbelopsis* species, suggest a largely conserved core gene content and functional repertoire within this genus. A substantial percentage of BUSCOs were identified within the annotated gene sets of all 3 *Umbelopsis* species, further confirming the completeness and reliability of the predicted gene models.

### GO analysis

**Fig. 2.**
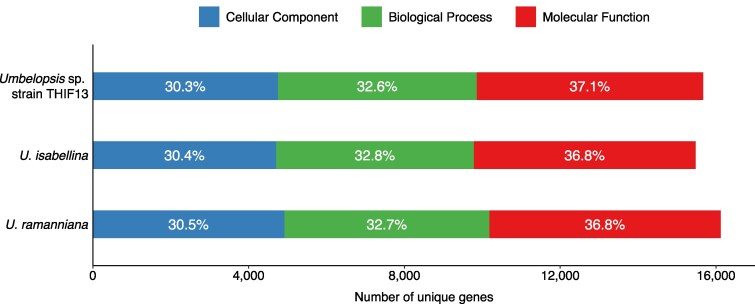
Distribution of gene ontology categories in *Umbelopsis* sp. strain THIF13, *U. isabellina*, and *U. ramanniana*: molecular function (red), cellular component (blue), and biological process (green).

**Fig. 3.**
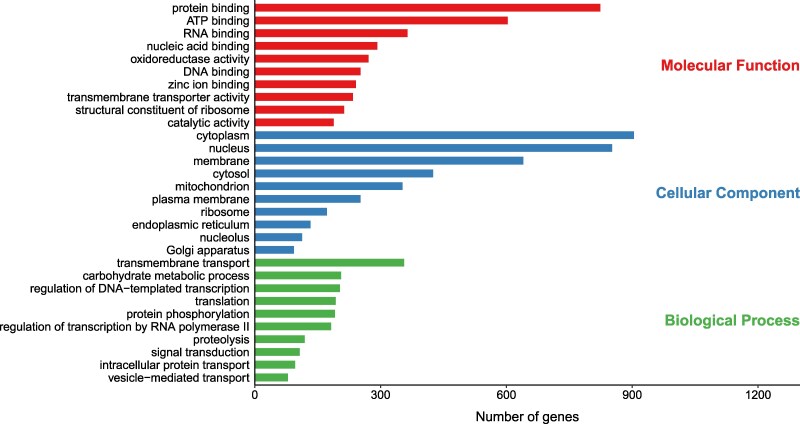
Distribution of GO categories in *Umbelopsis* sp. strain THIF13. The horizontal bar chart shows the number of genes assigned to GO terms within the 3 main GO domains. For each domain, the top 10 most abundant GO terms based on gene count are displayed.

To functionally categorize the predicted genes, GO analysis (Ashburner et al. 2000; Gene Ontology Consortium 2021) was performed for the *Umbelopsis* sp. strain THIF13 genome and compared with other species. The overall distribution of genes across the 3 main GO domains showed strong consistency among all 3 *Umbelopsis* species (Fig. 2). Further detailed analysis of the top 10 most abundant GO terms within each domain for *Umbelopsis* sp. strain THIF13 is presented in Fig. 3. Within the Molecular Function domain, highly represented terms included “protein binding,” “ATP binding,” and “RNA binding.” For Cellular Component, prominent terms comprised “cytoplasm,” “nucleus,” and “membrane,” indicating key cellular structures. In the Biological Process domain, “transmembrane transport,” “carbohydrate metabolic process,” and “regulation of transcription by RNA polymerase” were among the most frequently assigned terms. This strong conservation across major GO categories suggests a highly similar and fundamental functional repertoire among these *Umbelopsis* species, likely reflecting their shared core cellular processes and structures essential for fungal life.

### Analysis of CAZyme

**Fig. 4.**
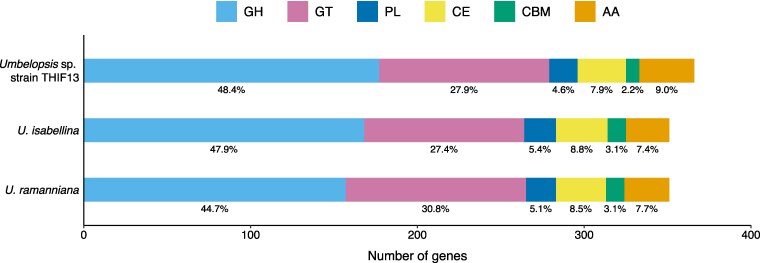
Distribution of CAZyme family repertoires in *Umbelopsis* sp. strain THIF13, *U. isabellina*, and *U. ramanniana*. Each bar is segmented by CAZyme class: glycoside hydrolases (GH, light blue), glycosyl transferases (GT, pink), polysaccharide lyases (PL, dark blue), carbohydrate esterases (CE, yellow), carbohydrate-binding modules (CBM, teal), and auxiliary activities (AA, orange).

The genes annotated as CAZymes (Cantarel et al. 2009; Lombard et al. 2014) were compared in the 3 *Umbelopsis* genomes (Fig. 4). Their CAZyme complements are highly conserved. Glycoside hydrolases (GHs) were the most abundant CAZyme class in all 3 species, with *Umbelopsis* sp. strain THIF13 having a slightly higher proportion, followed by Glycosyltransferases (GTs). Other CAZyme classes identified included auxiliary activities (AAs), carbohydrate esterases (CEs), polysaccharide lyases (PLs), and carbohydrate-binding modules (CBMs).

**Fig. 5.**
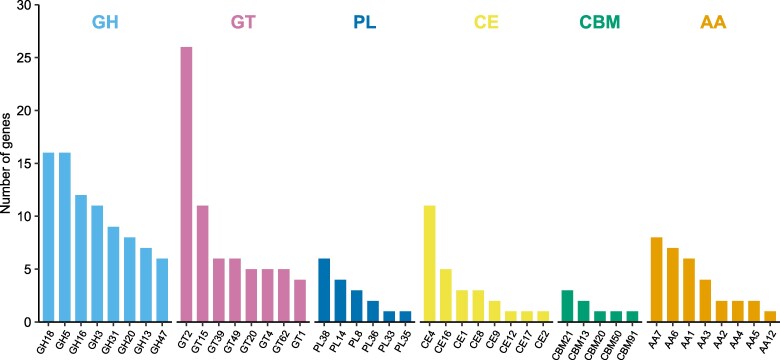
The bar chart displays the number of genes within individual CAZyme families, grouped by their broader classes. For each class, up to the 8 most abundant families are shown. Classes with fewer than 8 identified families display all detected families.

Delving into the specific CAZyme families (Fig. 5), the GH profile includes numerous families associated with deconstructing the polysaccharide components of plant cell walls and fungal biomass, such as GH5 (cellulases/hemicellulases) and GH18 (chitinases). Additionally, the genome contains other GH families, such as GH16 (comprising 12 enzymes in subfamilies 2, 3, 4, 18, 19, and 23; e.g. endo-*β*-1,3-glucanases and cell wall transglycosylases) (Viborg et al. 2019) and GH3 (e.g. β-glucosidases), typically involved in the hydrolysis of polysaccharides into assimilable mono- or oligo-saccharides. The genome also encodes enzymes known for the degradation of cellulose, including a GH6 family cellulase and an AA14 family lytic polysaccharide monooxygenase. The AA class is also well-represented, featuring AA1 (laccases), which are associated with the modification of lignin and other aromatic compounds, as well as AA3 (glucose–methanol–choline oxidoreductases), AA7 (oligosaccharide oxidases), and 2 AA2 ascorbate peroxidases (Levasseur et al. 2013). Additionally, GTs represent a major class, notably GT2 (e.g. chitin and cellulose synthases), known for its role in cell wall integrity and biosynthesis, which is essential for fungal growth. Finally, various PLs, CEs, and CBMs were also identified in the genome.

The prevalence of these enzymes, particularly GHs and GTs, suggests a strategy focused on polysaccharide degradation and cell wall synthesis, consistent with a saprotrophic lifestyle (Kubicek et al. 2014). Furthermore, the comprehensive CAZyme repertoire identified in *Umbelopsis* sp. strain THIF13, characterized by a broad array of families involved in the degradation of complex plant carbohydrates, supports its saprotrophic lifestyle and ability to thrive in lignocellulose-rich forest environments, consistent with the known ecology of *Umbelopsis* species (Osono 2011).

### KEGG pathway analysis

To further understand the metabolic and cellular capabilities of *Umbelopsis* sp. strain THIF13, its predicted genes were analyzed using the KEGG pathway database (Ogata et al. 1999; Kanehisa and Goto 2000; Du et al. 2014). The distribution of genes across Level B pathways, specifically those categorized within the major KEGG functional categories of “metabolism,” “genetic information processing,” “environmental information processing,” and “cellular processes,” was then compared with that of *U. isabellina* and *U. ramanniana* (Table 4). The KEGG Level B pathway profiles were highly similar across the 3 *Umbelopsis* species, reflecting conserved core metabolic and cellular functions. Among the various Level B pathways, “signal transduction” was the most highly represented category, indicating a substantial genetic investment in environmental sensing and cellular communication pathways, which are critical for fungal adaptation to diverse environments. Other prominent pathways included “carbohydrate metabolism,” “amino acid metabolism,” “transport and catabolism,” “lipid metabolism,” and “folding, sorting, and degradation.” The high representation of these pathways is consistent with their saprotrophic lifestyle, requiring robust machinery for nutrient acquisition, energy generation, and protein homeostasis from complex organic matter (Osono 2011; Kubicek et al. 2014). Furthermore, this metabolic potential likely supports lipid accumulation, a characteristic trait of *Umbelopsis* species, by providing essential precursors and energy.

**Table 4.** Summary of annotated genes assigned to KEGG in *Umbelopsis* sp. strain THIF13, *U. isabellina*, and *U. ramanniana*.

KEGG pathway (level B)	*Umbelopsis* sp. strain THIF13		*U. isabellina*		*U. ramanniana*	
Signal transduction	1,057	(17.49%)	1,005	(17.17%)	1017	(16.9%)
Transport and catabolism	551	(9.11%)	550	(9.4%)	570	(9.47%)
Carbohydrate metabolism	490	(8.11%)	443	(7.57%)	441	(7.33%)
Cell growth and death	475	(7.86%)	478	(8.17%)	463	(7.69%)
Translation	436	(7.21%)	423	(7.23%)	446	(7.41%)
Amino acid metabolism	405	(6.7%)	386	(6.6%)	411	(6.83%)
Folding, sorting, and degradation	323	(5.34%)	319	(5.45%)	326	(5.42%)
Lipid metabolism	306	(5.06%)	300	(5.13%)	339	(5.63%)
Glycan biosynthesis and metabolism	246	(4.07%)	225	(3.84%)	239	(3.97%)
Metabolism of cofactors and vitamins	223	(3.69%)	207	(3.54%)	224	(3.72%)
Replication and repair	214	(3.54%)	232	(3.96%)	227	(3.77%)
Energy metabolism	190	(3.14%)	182	(3.11%)	181	(3.01%)
Cellular community—eukaryotes	165	(2.73%)	147	(2.51%)	150	(2.49%)
Transcription	158	(2.61%)	161	(2.75%)	161	(2.68%)
Xenobiotics biodegradation and metabolism	128	(2.12%)	146	(2.49%)	149	(2.48%)
Cell motility	123	(2.03%)	111	(1.9%)	119	(1.98%)
Metabolism of other amino acids	118	(1.95%)	113	(1.93%)	113	(1.88%)
Nucleotide metabolism	103	(1.7%)	105	(1.79%)	106	(1.76%)
Biosynthesis of other secondary metabolites	82	(1.36%)	78	(1.33%)	78	(1.3%)
Chromosome	63	(1.04%)	60	(1.03%)	59	(0.98%)
Metabolism of terpenoids and polyketides	59	(0.98%)	59	(1.01%)	59	(0.98%)
Signaling molecules and interaction	50	(0.83%)	48	(0.82%)	60	(1%)
Information processing in viruses	34	(0.56%)	35	(0.6%)	38	(0.63%)
Cellular community—prokaryotes	33	(0.55%)	27	(0.46%)	29	(0.48%)
Membrane transport	13	(0.22%)	12	(0.21%)	13	(0.22%)
Total annotated genes	6,045		5,852		6,018	

### Phylogenetic analysis

**Fig. 6.**
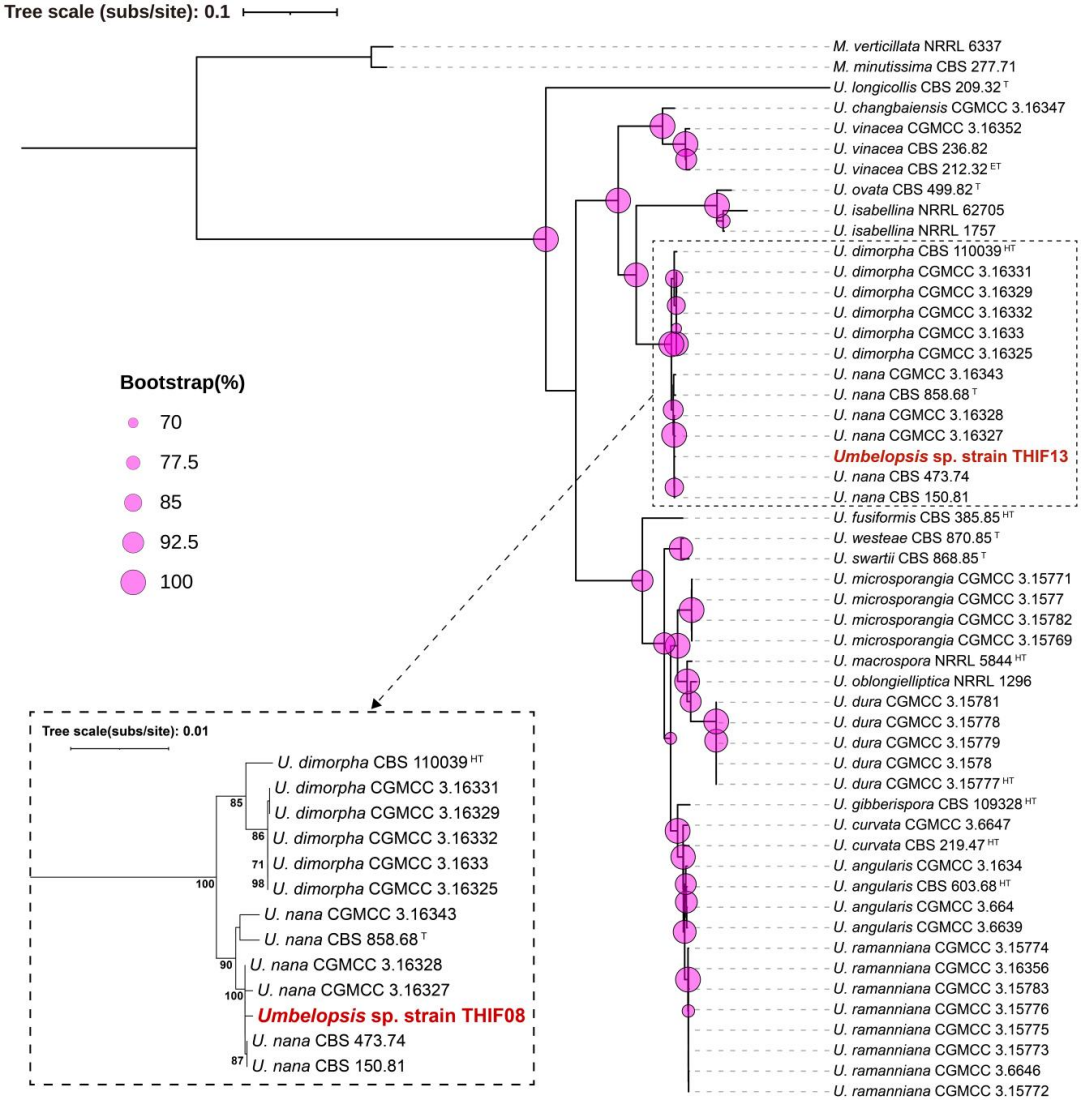
Phylogenetic tree of 49 *Umbelopsis* isolates and 2 *Mortierella* outgroups inferred from a concatenated alignment of 6 loci (SSU rDNA, ITS, LSU rDNA, *ACT*, *MCM7*, and *COX1*; 5,121 bp). The tree was reconstructed by maximum likelihood in IQ-TREE 2. Node support was assessed with 1,000 ultrafast bootstrap replicates; values on the main tree are represented by scaled circles, while values on the inset are shown as percentages. The inset shows a magnified view of the clade containing *Umbelopsis* sp. strain THIF13 (highlighted in red). Scale bars indicate substitutions per site. The isolate *U. dura* CGMCC 3.1578 was excluded because its sequence was identical to that of *U. dura* CGMCC 3.15777. HT, ex-holotype strain; ET, ex-epitype strain; T, ex-type strain.

In the previous study, initial ITS region sequencing and BLAST homology searches against GenBank failed to resolve the species identity of *Umbelopsis* sp. strain THIF13 (Masaki et al. 2016). Therefore, in this study, following Wang et al. (2022), 6 loci were selected for phylogenetic inference (SSU rDNA, ITS, LSU rDNA, *ACT*, *MCM7*, and *COX1*) (Table 1). Sequences from 50 *Umbelopsis* strains and 2 *Mortierella* species as outgroups were concatenated into a 5,121 bp matrix (ITS: 605 bp, LSU: 1,020 bp, SSU: 1,097 bp, *ACT*: 868 bp, *MCM7*: 983 bp, and *COX1*: 548 bp) and analyzed by maximum likelihood in IQ-TREE 2. In the resulting tree (Fig. 6), the overall tree topology and the robust support for major clades were largely consistent with previous multilocus phylogenetic analyses of *Umbelopsis* (Wang et al. 2022; Hou et al. 2024). Notably, *Umbelopsis* sp. strain THIF13 was clearly resolved within the *Umbelopsis nana* clade, a placement strongly supported by both ultrafast bootstrap (UFBoot = 90%) and SH-aLRT (93%) tests. This clade also contains the ex-type strain of *U. nana* (CBS 858.68, originally described as *Mortierella alba*) (Von Arx 1982). Pairwise p-distances among the 7 *U. nana* strains, including strain THIF13, were below the detection threshold (0 to 1 × 10⁻⁶), indicating near-identity across the concatenated loci. The split separating *U. nana* from *U. dimorpha* received 100% ultrafast bootstrap support. The BI analysis (Supplementary Fig. 2) yielded a congruent topology for all major clades, confirming the placement of *Umbelopsis* sp. strain THIF13 within the *U. nana* clade with the highest posterior probability (PP = 1.00). However, the branching order among closely related strains within the *U. nana* clade differed significantly between the ML and BI analyses. While the ML tree resolved the clade via sequential bifurcations, the BI tree defined this region as a polytomy where 4 branches diverge from a single node. This inconsistency confirms that the low genetic divergence (*P*-distances ≈ 0) among these *U. nana* strains prevents robust resolution of the internal topology. These results collectively confirm the assignment of strain THIF13 to *U. nana*. Following this identification, the strain has been deposited in the NBRC culture collection under the accession number *U. nana* NBRC 117090. Hereafter, we refer to this organism as *U. nana* strain THIF13 throughout the remainder of this manuscript. This genome represents the first publicly available genome sequence for *U. nana*.

## Conclusion

In this study, the first high-quality genome sequence of *Umbelopsis nana* strain THIF13 (NBRC 117090), whose species identity was clearly established through multilocus phylogenetic analysis, was successfully generated and characterized. Comprehensive functional annotation, including comparative analyses of Gene Ontology terms, CAZymes, and KEGG pathways against other *Umbelopsis* species, revealed a robust and versatile metabolic repertoire, which is broadly conserved among *Umbelopsis* species. The genomic resource presented herein substantially expands the currently limited genomic data for the *Umbelopsis* genus. This provides a critical foundation for future research, enabling fine-scale comparative genomic studies to identify specific genetic determinants of unique biological traits of *U. nana* (such as COS production by strain THIF13) that are not captured by broad functional annotations, as well as the exploration of its biotechnological potential.

## Supplementary Material

jkag022_Supplementary_Data

## Data Availability

The data generated for this study were submitted to the DNA Data Bank of Japan (DDBJ) and are publicly available through the International Nucleotide Sequence Database Collaboration (INSDC). All data are accessible under BioProject accession number PRJDB25744 and BioSample accession number SAMD00919913. The raw sequencing data are registered in the DDBJ Sequence Read Archive under accession number DRA021410. The accession numbers for the individual contigs of the genome assembly are BAAHSD010000001-BAAHSD010000018. The accession numbers for the genes used in the phylogenetic analysis are listed in Table 1. The computational scripts used for genome assembly, annotation, and phylogenetic analysis are available as Supplementary data. Supplemental material available at G3 online.
