# Supplementary material for: First high-quality genome assembly of Umbelopsis nana isolated from forest soil
Source: G3 (Bethesda). 2026 Jan 29;16(4):jkag022. doi: 10.1093/g3journal/jkag022 (PMC13042297; doi:10.1093/g3journal/jkag022)
Supplement: jkag022_Supplementary_Data [file jkag022_supplementary_data.zip › Supplemental_Figures_G3-2025-406498.pdf]

A

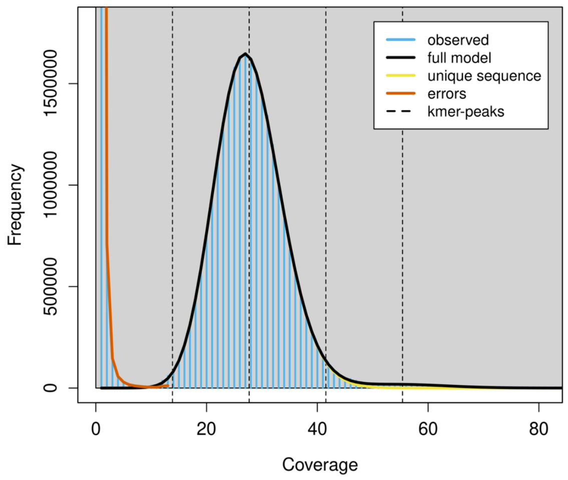

B

	min	max
Homozygous (aa)	99.97%	100%
Heterozygous (ab)	0%	0.03%
Genome Haploid Length	31,156,198 bp	31,212,235 bp
Genome Repeat Length	6,801,796 bp	6,814,030 bp
Genome Unique Length	24,354,402 bp	24,398,205 bp
Model Fit	79.29%	98.38%
Read Error Rate	0.09%	0.09%

Supplementary Figure S1 GenomeScope analysis profile of *Umbelopsis* sp. strain THIF13. (A) K-mer spectrum profile (k=21) generated from PacBio HiFi reads. (B) Summary table of genome characteristics estimated by GenomeScope.

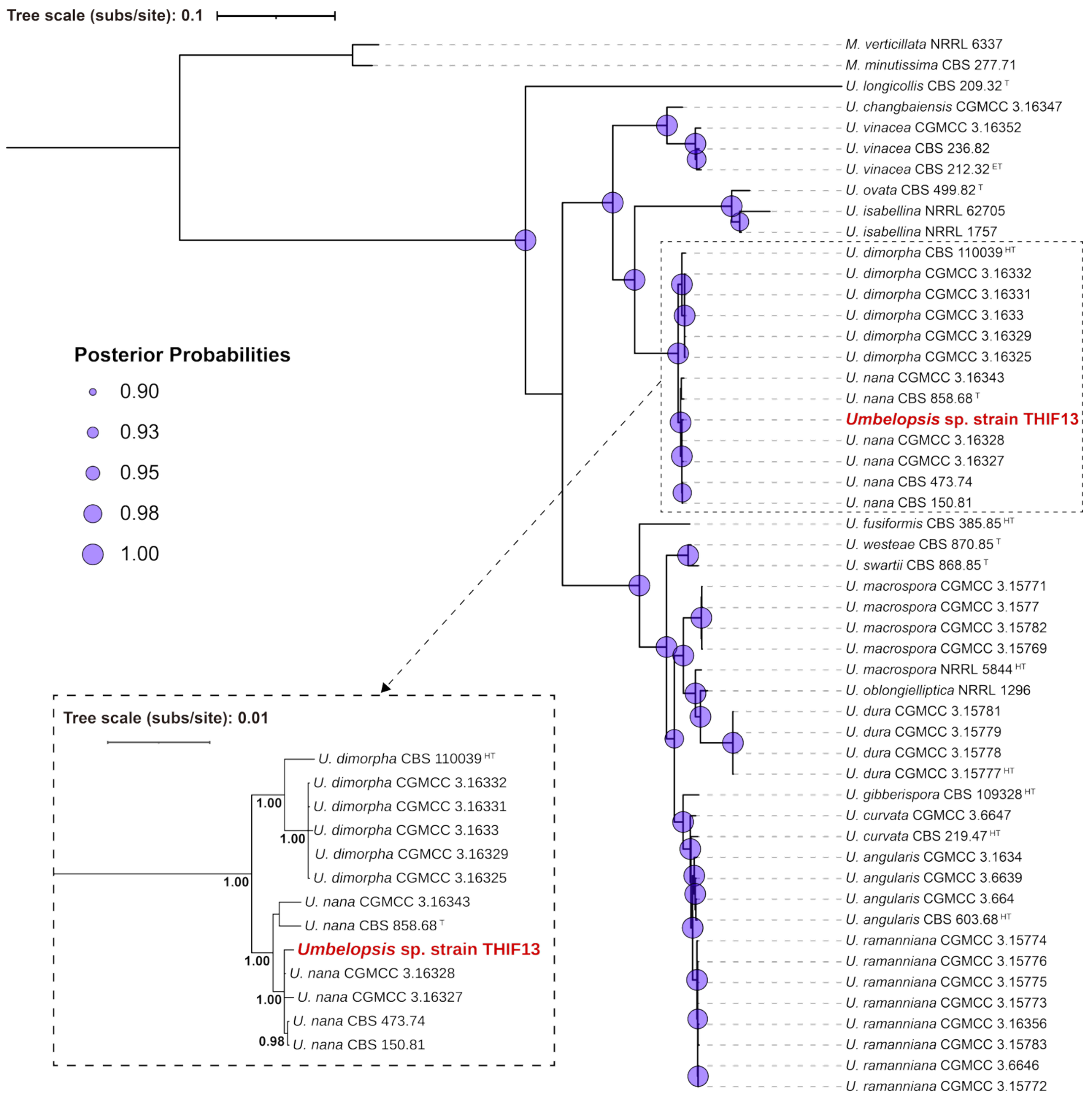

Supplementary Figure S2 Bayesian phylogenetic tree of *Umbelopsis* species inferred from a concatenated six-locus dataset (SSU rDNA, ITS, LSU rDNA, *ACT*, *MCM7* and *COX1*; 5,121 bp). The analysis was conducted in MrBayes, running for 10 million generations and sampling every 1,000 generations. Node support is represented by Posterior Probabilities; values on the main tree are represented by scaled circles, while values on the inset are shown as numerical values. Posterior Probabilities were calculated after discarding the first 25% of samples as burn-in. The tree was rooted with Mortierella species as the outgroup. Scale bars indicate substitutions per site. HT, ex-holotype strain; ET, ex-epitype strain; and T, ex-type strain.
