# Supplementary material for: First high-quality genome assembly of Umbelopsis nana isolated from forest soil
Source: G3 (Bethesda). 2026 Jan 29;16(4):jkag022. doi: 10.1093/g3journal/jkag022 (PMC13042297; doi:10.1093/g3journal/jkag022)
Supplement: jkag022_Supplementary_Data [file jkag022_supplementary_data.zip › Supplemental_Material_Legends_G3-2025-406498.docx]

**Supplementary data**

Supplementary File 1: SF1_hifiasm_script.txt

Script for genome assembly using hifiasm.

Supplementary File 2: SF2_circlator_script.txt

Script for genome circularization using Circlator.

Supplementary File 3: SF3_tidk_script.txt

Script for telomere identification using tidk.

Supplementary File 4: SF4_RepeatModeler_RepeatMasker_script.txt

Scripts for repeat modeling and masking using RepeatModeler and RepeatMasker.

Supplementary File 5: SF5_Funannotate_script.txt

Scripts for functional annotation of the genome using Funannotate and eggNOG-mapper.

Supplementary File 6: SF6_Phylogenetic_analysis_script.sh

Shell script for performing phylogenetic analysis, including sequence alignment with MAFFT, trimming with trimAl, and tree construction with IQ-TREE 2.

Supplementary Figures: Supplementary_Figures.docx

This file includes Supplementary Figure S1 and Supplementary Figure S2.
